# Impact of seasons on elderly patients with esophageal squamous cell carcinoma following esophagectomy: a propensity score matching analysis

**DOI:** 10.1007/s12672-025-03009-w

**Published:** 2025-06-23

**Authors:** Kexun Li, Simiao Lu, Changding Li, Jie Mao, Huan Zhang, Kangning Wang, Guangyuan Liu, Yunchao Huang, Yongtao Han, Xuefeng Leng, Lin Peng

**Affiliations:** 1 Department of Thoracic Surgery, Sichuan Cancer Hospital & Institute, Sichuan Cancer Center, School of Medicine, University of Electronic Science and Technology of China, Chengdu. No. 55, Section 4, South Renmin Road, Chengdu, 610041 Sichuan China; 2 Department of Thoracic Surgery I, Third Affiliated Hospital of Kunming Medical University (Yunnan Cancer Hospital, Yunnan Cancer Center), Kunming, China

**Keywords:** Elderly ESCC patients, Seasons, Overall survival, Disease-free survival, Postoperative complications

## Abstract

**Background:**

Esophageal squamous cell carcinoma (ESCC) is prevalent in East Asia, with elderly patients facing unique postoperative challenges. This study examines the impact of seasonal variations on postoperative complications and survival in elderly patients undergoing esophagectomy for ESCC.

**Methods:**

This retrospective cohort study was conducted using data from the Sichuan Cancer Hospital & Institute Esophageal Cancer Case Management Database. Elderly patients (≥ 70 years) with thoracic ESCC who underwent esophagectomy between May 2016 and August 2021 were included. Patients were stratified into four seasonal groups: Winter (December–February), Spring (March–May), Summer (June–August), and Autumn (September–November). Primary outcomes included overall survival (OS), disease-free survival (DFS). Secondary outcomes assessed postoperative complications using the Clavien-Dindo classification.

**Results:**

A total of 469 elderly ESCC patients were included. The median overall survival was 51.6 months, with no significant differences in OS or DFS across the four seasonal groups. Restricted Mean Survival Time (RMST) and Restricted Mean Disease-Free Survival Time (RMDFST) analyses also showed no significant seasonal variations. The Summer group had a significantly higher incidence of hydrothorax compared to other groups (p < 0.05).

**Conclusions:**

Seasonal variations influence specific short-term postoperative complications but do not significantly impact long-term survival in elderly ESCC patients undergoing esophagectomy.

## Introduction

Esophageal cancer (EC) is a major global health concern, with over half of the cases concentrated in East Asia, particularly in countries like China and Japan [1, 2]. Unlike Western regions such as Europe and the United States, where adenocarcinoma is more common, esophageal squamous cell carcinoma (ESCC) predominates in East Asia [1–4]. In recent years, treatment for ESCC, particularly in locally advanced cases, has evolved into a multimodal approach. Neoadjuvant therapies, including chemotherapy, radiotherapy, and more recently, immunotherapy, have been integrated with surgery to improve clinical outcomes [5–8]. These treatments can reduce tumor size, increase resectability, and lower the risk of recurrence, ultimately improving overall survival rates [9–12]. Even after surgery, many patients require additional adjuvant treatments based on pathological risk factors. Postoperative chemotherapy, radiotherapy, and immunotherapy are crucial in reducing the chance of ESCC recurrence, thereby enhancing long-term survival [5, 13–15].

While the global incidence of esophageal cancer has been showing a downward trend, the aging population has resulted in a growing number of elderly patients being diagnosed with the disease [16–19]. This demographic shift has led to an increasing focus on the unique challenges that older patients face, particularly in managing the risks associated with complex procedures like esophagectomy. Elderly individuals often present with reduced physical reserves and coexisting medical conditions, making them more susceptible to postoperative complications and poorer outcomes [20–23].

Environmental factors, including seasonal variations, have been shown to influence human metabolism, protein functionality, and microbial activity, all of which can significantly affect recovery and postoperative outcomes [24–28]. Given that seasonal changes can result in substantial temperature differences between winter, spring, summer, and fall, it is plausible that the time of year in which surgery is performed may influence complication rates, recovery times, or even survival outcomes in patients undergoing esophagectomy. However, the impact of seasonal variation on the prognosis of elderly ESCC patients following surgery has not been extensively studied.

This study aims to investigate whether seasonal differences have an effect on postoperative complications and long-term survival in elderly patients with ESCC undergoing esophagectomy. By analyzing patient outcomes across distinct seasons, this research seeks to identify potential trends that could improve clinical management for this vulnerable population.

## Methods

### Study design and patient selection

This retrospective cohort study was conducted using the Sichuan Cancer Hospital & Institute Esophageal Cancer Case Management Database (SCCH-ECCM Database). Elderly patients (≥ 70 years old) diagnosed with thoracic esophageal squamous cell carcinoma (ESCC) and undergoing esophagectomy between May 2016 and August 2021 were included. Eligible patients were required to have histologically confirmed ESCC without evidence of distant metastasis based on clinical imaging (e.g., CT, B-ultrasonography). Patients were excluded if they had non-thoracic esophageal tumors, other histological types of esophageal cancer, incomplete clinical data, or lacked follow-up information. The final follow-up date was December 20, 2023.

### Grouping and outcome measures

Patients were stratified into seasonal groups based on the time of surgery: Winter Group (December-February), Spring Group (March–May), Summer Group (June–August), and Autumn Group (September–November). Primary outcomes included overall survival (OS), disease-free survival (DFS), Restricted Mean Survival Time (RMST) and Restricted Mean Disease-Free Survival Time (RMDFST). Secondary outcomes evaluated postoperative complication rates across seasons using standardized criteria. OS was calculated from the date of surgery to the date of death or last follow-up, while DFS was measured from surgery to the first documented recurrence, death, or last follow-up. Additionally, RMST and RMDFST were calculated to assess survival outcomes over a restricted time horizon. Tumor staging adhered to the 8th edition UICC/AJCC TNM classification [5], and pathological diagnoses were validated by two independent pathologists.

### Criteria and characteristics of the adverse events

Postoperative complications were classified using the Clavien-Dindo system, with grades III and IV representing severe complications requiring invasive interventions or ICU management. Perioperative mortality was defined as in-hospital death or death within 30 days of surgery [29–31]. Patients’ BMI was grouped into underweight (< 18.5 kg/m^2^), normal weight (18.5–24.9 kg/m^2^), and overweight/obese (≥ 25 kg/m^2^) categories, per WHO guidelines [32, 33].

### Statistical analyses

Baseline characteristics of the study population were compared across seasonal groups using chi-square tests for categorical variables. Survival outcomes, including OS and DFS, were analyzed using Kaplan–Meier methods, and differences between seasonal groups were assessed with log-rank tests. To quantify the survival time within a restricted period, RMST and RMDFST were calculated for each group. RMST and RMDFST were estimated by integrating the survival and disease-free survival curves from time 0 to a predefined time horizon τ, providing a summary measure of survival within this restricted period. Cox proportional hazards regression models were used to identify independent prognostic factors for OS and DFS, with adjustments for potential confounders such as age, sex, tumor stage, BMI, and comorbidities. A two-sided p-value < 0.05 was considered statistically significant. To account for potential confounding, PSM was performed using a 1:1 nearest-neighbor algorithm without replacement. The matching process balanced key variables, including age, tumor stage, KPS score, and comorbidity index, between seasonal groups. This approach aimed to reduce bias in estimating the treatment effect of seasonal surgery timing on survival outcomes. All statistical analyses were performed using SPSS version 26.0 and RStudio version 4.3.0.

### Ethical considerations

Ethical approval was obtained from the Ethics Committee for Medical Research and New Medical Technology at Sichuan Cancer Hospital (Approval No. SCCHEC-02–2024-191). As a retrospective study, patient consent was waived, but confidentiality and data protection protocols were strictly maintained in accordance with the Declaration of Helsinki (2013 revision). The need for informed consent was waived by Ethics Committee for Medical Research and New Medical Technology of Sichuan Cancer Hospital (SCCHEC-02–2024-191).

## Results

### Demographic data

A total of 469 patients were included in the study. Among these, 27 patients (5.76%) were aged 80 years or older. Preoperative assessments showed that 373 patients (79.5%) had locally advanced disease, with cT3 or cT4 stage based on preoperative esophageal ultrasound and CT scans. Only 81 patients (17.3%) were considered without lymph node (LN) metastasis before surgery. Of the 469 patients, 358 (76.3%) underwent minimally invasive esophagectomy (MIE) (Table 1).

**Table 1:** Demographic characteristics of the groups

Characteristic	Total	Spring (n = 147)	Summer (n = 116)	Autumn (n = 106)	Winter (n = 100)
Sex					
Male	375	121(82.31%)	88(75.86%)	89(83.96%)	77(77.00%)
emale	94	26 (17.69%)	28(24.14%)	17(16.04%)	23(23.00%)
Age, years					
median (range)	73(70–88)	73(70–88)	73(70–81)	73(70–81)	73(70–83)
< 80	442	139(94.56%)	112(96.55%)	97(91.51%)	94(94.00%)
≥ 80	27	8(5.44%)	4(3.45%)	9(8.49%)	6(6.00%)
Smoking					
Yes	241	78(53.06%)	54(46.55%)	62(58.49%)	47(47.00%)
No	228	69(46.94%)	62(53.45%)	44(41.51%)	53(53.00%)
Alcohol					
Yes	245	86(58.50%)	56(48.28%)	56(52.83%)	47(47.00%)
No	224	61(41.50%)	60(51.72%)	50(47.17%)	53(53.00%)
Tumor location					
Upper	61	17(11.56%)	14(12.07%)	14(13.21%)	16(16.00%)
Middle	207	59(40.14%)	47(40.52%)	55(51.89%)	46(46.00%)
Lower	201	71(48.30%)	55(47.41%)	37(34.91%)	38(38.00%)
BMI					
Low	34	9(6.12%)	16(13.79%)	5(4.72%)	4(4.00%)
Normal	360	112(76.19%)	87(75.00%)	87(82.08%)	74(74.00%)
High	75	26(17.69%)	13(11.21%)	14(13.21%)	22(22.00%)
KPS score					
≤ 80	119	41(27.89%)	39(33.62%)	16(15.09%)	23(23.00%)
≥ 90	350	106(72.11%)	77(66.38%)	90(84.91%)	77(77.00%)
Surgical approach					
McKeown	407	128(87.07%)	96(82.76%)	95 (89.62%)	88(88.00%)
Lovr-Lewis	62	19(12.93%)	20(17.24%)	11(10.38%)	12(12.00%)
cT stage					
T1	29	12(8.16%)	5(4.31%)	7(6.60%)	5(5.00%)
T2	67	24 (16.33%)	19 (16.38%)	13(12.26%)	11(11.00%)
T3	324	93(63.27%)	80(68.97%)	74(69.81%)	77(77.00%)
T4	49	18(12.24%)	12(10.34%)	12(11.32%)	7(7.00%)
cN stage					
N0	81	28(19.05%)	18(15.52%)	17(16.04%)	18(18.00%)
N1	282	89(60.54%)	68(58.62%)	62(58.49%)	63(63.00%)
N2	100	28(19.05%)	30(25.86%)	24(22.64%)	18(18.00%)
N3	6	2 (1.36%)	0(0.00%)	3(2.83%)	1(1.00%)
cTNM Stage					
I	27	12(8.16%)	5(4.31%)	6(5.66%)	4(4.00%)
II	100	38(25.85%)	25(21.55%)	19(17.92%)	18(18.00%)
III	286	76(51.70%)	74(63.79%)	66(62.26%)	70(70.00%)
IV	56	21(14.29%)	12(10.34%)	15(14.15%)	8(8.00%)
Neoadjuvant therapy					
Yes	72	21(14.29%)	23(19.83%)	16(15.09%)	12(12.00%)
No	397	126(85.71%)	93(80.17%)	90(84.91%)	88(88.00%)

KPS: Karnofsky Performance Status; PSM: propensity score matching; TNM: tumor, node, metastasis

In terms of pathological features, 164 patients (35.0%) exhibited lymphovascular invasion, and 204 patients (43.5%) had nerve invasion. A total of 452 patients (96.4%) successfully underwent R0 resection, indicating complete surgical resection with no residual cancer at the resection margin (Table 2).

**Table 2:** Demographic characteristics after surgery

Characteristic	Total	Spring (n = 147)	Summer (n = 116)	Autumn (n = 106)	Winter (n = 100)
Lymphovascular invasion					
Yes	164	51(34.69%)	43(37.07%)	38(35.85%)	32(32.00%)
No	305	96(65.31%)	73(62.93%)	68(64.15%)	68(68.00%)
Nerve invasion					
Yes	204	61(41.50%)	44(37.93%)	56(52.83%)	43(43.00%)
No	265	86(58.50%)	72(62.07%)	50(47.17%)	57(57.00%)
Complete resection					
R0	452	141(95.92%)	112(96.55%)	101(95.28%)	98(98.00%)
R1/R2	17	6(4.08%)	4(3.45%)	5(4.72%)	2(2.00%)
Pathological differentiation grade					
Moderate or Well G1-2	328	99(67.35%)	86(74.14%)	75(70.75%)	68(68.00%)
Poor or undifferentiated G3-4	141	48(32.65%)	30(25.86%)	31(29.25%)	32(32.00%)
Neoadjuvant therapy					
Yes	72	21(14.29%)	23(19.83%)	16(15.09%)	12(12.00%)
No	397	126(85.71%)	93(80.17%)	90(84.91%)	88(88.00%)
Pathological T stage					
T0	12	2(1.36%)	5(4.31%)	2(1.89%)	3(3.00%)
T1	73	26(17.69%)	21(18.10%)	13(12.26%)	13(13.00%)
T2	94	29(19.73%)	28(24.14%)	20(18.87%)	17(17.00%)
T3	269	83(56.46%)	58(50.00%)	66(62.26%)	62(62.00%)
T4	21	7(4.76%)	4(3.45%)	5(4.72%)	5(5.00%)
Pathological N stage					
N0	244	73(49.66%)	61(52.59%)	57(53.77%)	53(53.00%)
N1	135	45(30.61%)	29(25.00%)	30(28.30%)	31(31.00%)
N2	68	23(15.65%)	18(15.52%)	13(12.26%)	14(14.00%)
N3	22	6(4.08%)	8(6.90%)	6(5.66%)	2(2.00%)
Pathological 8th TNM Stage					
I	88	27(18.37%)	29(25.00%)	17(16.04%)	15(15.00%)
II	154	45(30.61%)	35(30.17%)	37(34.91%)	37(37.00%)
III	193	63(42.86%)	44(37.93%)	43(40.57%)	43(43.00%)
IV	34	12(8.16%)	8(6.90%)	9(8.49%)	5(5.00%)
Died in 30 days	3	0(0.00%)	0(0.00%)	3(2.83%)	0(0.00%)
Died in 90 days	14	2(1.36%)	1(0.86%)	7(6.60%)	4(4.00%)
Adverse events (Clavien–Dindo)					
0-II	234	71(48.30%)	57(49.14%)	52(49.06%)	54(54.00%)
III-IV	233	76(51.70%)	59(50.86%)	52(49.06%)	46(46.00%)
V	2	0(0.00%)	0(0.00%)	2(1.89%)	0(0.00%)

KPS: Karnofsky Performance Status; PSM: propensity score matching; TNM: tumor, node, metastasis

**Fig. 1:**
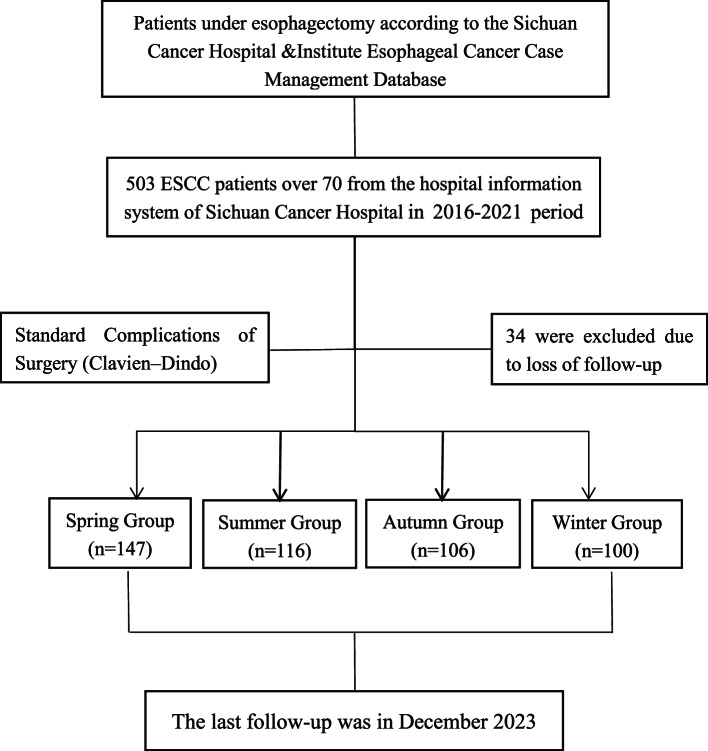
CONSORT diagram of patient selection

The patients were distributed across the four seasonal groups as follows: 147 patients (31.3%) were in the Spring Group, 116 patients (24.7%) in the Summer Group, 106 patients (22.6%) in the Autumn Group, and 100 patients (21.3%) in the Winter Group (Fig. 1).

### Overall survival and disease free survival

**Fig. 2:**
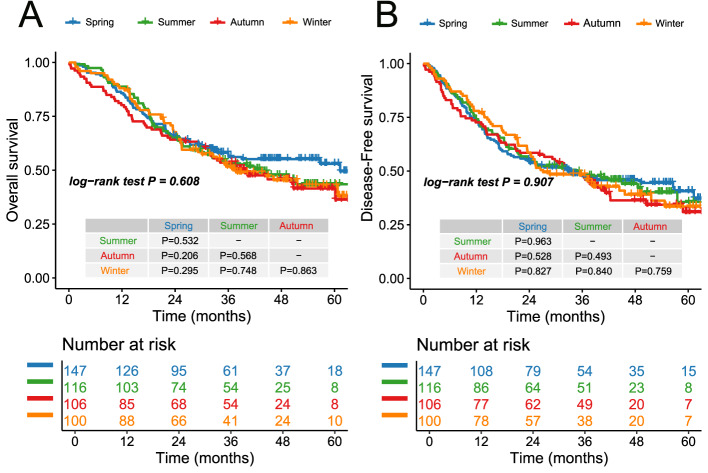
**A** Overall survival curves of participants in 4 groups; **B** Disease-free survival curves of participants in 4 groups

Among the 469 patients, the median follow-up duration was 47.5 months, with a median OS time of 51.6 months (95% CI 38.10–65.10). Across the four seasonal groups, no statistically significant differences were observed in OS (p-values ranged from 0.206 to 0.863). The Spring Group demonstrated the longest median OS at 61.3 months (95% CI 40.5–82.0), with 1-, 3-, and 5-year OS rates of 86%, 57%, and 52%, respectively. The Summer Group had a median OS of 43.1 months (95% CI 28.5–57.7), and the 1-, 3-, and 5-year OS rates were 89%, 53%, and 42%. The Autumn Group showed a median OS of 40.2 months (95% CI 25.6–54.8), with OS rates at 1, 3, and 5 years of 80%, 54%, and 40%. Similarly, the Winter Group’s median OS was 40.2 months (95% CI 22.7–52.8), with 1-, 3-, and 5-year OS rates of 88%, 51%, and 43%. Despite the observed numerical differences, no significant survival advantage was linked to seasonal timing of surgery (Fig. 2A).

DFS also showed no significant differences among the seasonal groups, with p-values ranging from 0.493 to 0.963. The Spring Group had a median DFS time of 33.0 months (95% CI 13.1–52.9), while the Summer Group exhibited a slightly longer median DFS of 34.6 months (95% CI 17.8–51.4). The Autumn Group’s median DFS was 33.5 months (95% CI 26.6–40.4), and the Winter Group had the shortest DFS at 27.1 months (95% CI 15.8–38.4). Despite these variations, no significant seasonal differences in DFS were identified, suggesting that the timing of surgery within a given season did not notably influence recurrence-free outcomes for elderly patients with ESCC (Fig. 2B).

### Restricted mean survival time and restricted mean disease-free survival time

**Fig. 3:**
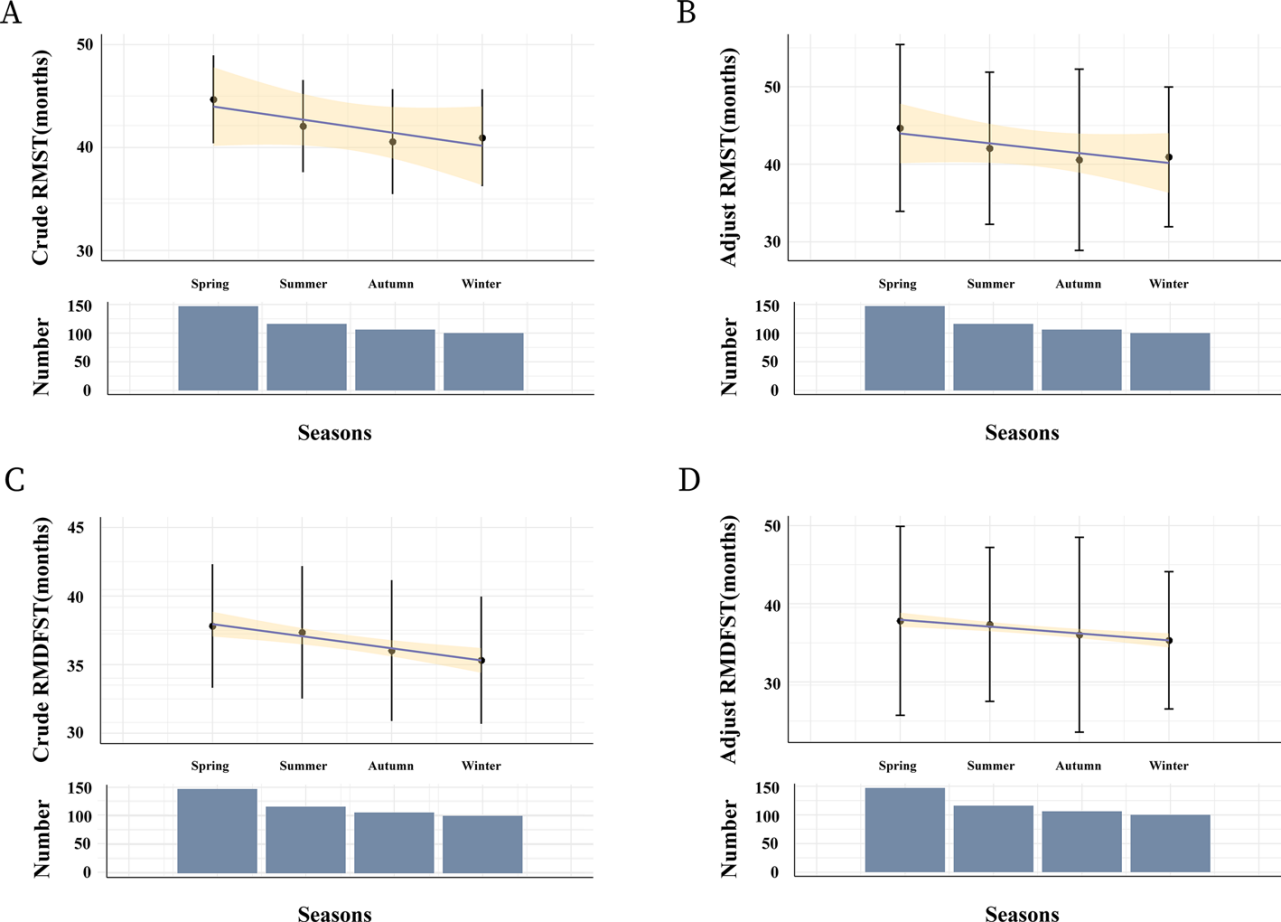
Restricted mean survival time (RMST) and Restricted Mean Disease-Free Survival Time (RMDFST) estimates patients. **A** Crude RMST estimates different patients; **B** Adjust RMST estimates different patients; **C** Crude RMDFST estimates different patients; **D** Adjust RMDFST estimates different patients

Analysis of RMST revealed that all four subgroups exhibited RMST values exceeding 40 months, demonstrating a consistent trend across seasons. The Spring Group demonstrated the longest RMST at 44.7 months (Crude 95% CI: 40.42–48.95; Adjusted 95% CI 33.93–55.43). The Summer Group had an RMST of 42.1 months (Crude 95% CI 37.60–46.56; Adjusted 95% CI 32.26–51.87), followed by the Winter Group at 41.0 months (Crude 95% CI 36.25–45.65; Adjusted 95% CI 31.95–49.96), and the Autumn Group at 40.6 months (Crude 95% CI 35.48–45.67; Adjusted 95% CI 28.91–52.25) (Fig. 3A, B).

Similarly, analysis of RMDFST showed that all four seasonal groups had RMDFST values above 40 months, maintaining a comparable trend. The Spring Group showed the longest RMDFST at 37.8 months (Crude 95% CI 33.32–42.32; Adjusted 95% CI 25.73–49.90), followed by the Summer Group at 37.4 months (Crude 95% CI 32.53–42.18; Adjusted 95% CI 27.50–47.20). The Autumn Group had an RMDFST of 36.0 months (Crude 95% CI: 30.90–41.16; Adjusted 95% CI 23.57–48.49), and the Winter Group had the shortest RMDFST at 35.3 months (Crude 95% CI 30.69–39.96; Adjusted 95% CI 26.55–44.11) (Fig. 3C, D).

### Short-term outcomes and adverse events (Clavien-Dindo, 2009)

Of the 469 patients included in the study, 233 (49.7%) experienced Clavien-Dindo grade III-IV postoperative complications. Three patients died within 30 days of surgery, and 14 patients died within 90 days. A breakdown by season reveals the following rates of Clavien-Dindo grade III-IV complications: Spring Group, 76 patients (51.70%); Summer Group, 59 patients (50.86%); Autumn Group, 52 patients (49.06%); and Winter Group, 46 patients (46.00%). Two patients died during the perioperative period (Tables 2 and  3).

**Table 3:** Adverse events (Clavien–Dindo ≥ III, 2009)

Adverse events	Spring (n = 147)			Summer (n = 116)			Autumn (n = 106)			Winter (n = 100)		
	III	IV	V	III	IV	V	III	IV	V	III	IV	V
Anastomotic stenosis	25(17.01)			16(13.79)			14(13.21)			16(16.00)	1 (1.00)	
Anastomotic leakage	8(5.44)	4(2.72)		11(9.48)	6(5.17)		15(14.15)	4(3.77)	1 (0.94)	9(9.00)	6(6.00)	
Pulmonary infection	15(10.20)	15(10.20)		17(14.66)	15(12.93)		9(8.49)	13(12.26)	2 (1.89)	10(10.00)	6(6.00)	
Hydrothorax	19(12.93)			32(27.59)			16(15.09)			14(14.00)		
Respiratory failure	1(0.68)	11(7.48)		1(0.86)	7(6.03)		1 (0.94)	12(11.32)	2 (1.89)		7(7.00)	
Heart failure	7(4.76)	2(1.36)		5(4.31)	2(1.72)		3(2.83)	3(2.83)	1 (0.94)	2(2.00)	1(1.00)	
Postoperative hoarseness	3(2.04)	2(1.36)		3(2.59)			6(5.66)	1 (0.94)		6(6.00)		
Postoperative bleeding	4(2.72)	2(1.36)		4(3.45)	2(1.72)		2 (1.89)	3(2.83)		7(7.00)		
Arrhythmia	8(5.44)	1(0.68)		7(6.03)	1(0.86)		2 (1.89)	2 (1.89)		4(4.00)	1(1.00)	
Pneumothorax	13(8.84)			5(4.31)			9(8.49)			3(3.00)		
Abnormal liver function	5(3.40)	1(0.68)		4(3.45)			3(2.83)	1 (0.94)		1(1.00)		
Fever	7(4.76)			5(4.31)			3(2.83)			1(1.00)		
Pulmonary atelectasis	5(3.40)			5(4.31)			1 (0.94)			1(1.00)		
Suspected anastomotic leakage	1(0.68)			2(1.72)								
Chylous fistula	2(1.36)			3(2.59)	3(2.59)			2 (1.89)		2(2.00)		
ARDS		1(0.68)			3(2.59)			3(2.83)				
Pyothoraxs	2(1.36)	1(0.68)			1(0.86)							
Wound infection	1(0.68)				1(0.86)		2 (1.89)	1 (0.94)				
Pulmonary embolism							1 (0.94)	1 (0.94)	1 (0.94)			
Delirium		1(0.68)								1(1.00)		
Thrombosis	3(2.04)			2(1.72)						1(1.00)		
Ketosis					1(0.86)							
Renal injury	2(1.36)			3(2.59)	1(0.86)			1 (0.94)				
Tracheal injury					2(1.72)				1 (0.94)			
Cerebral infarction	1(0.68)											
Gastric perforation		1(0.68)										
Diaphragmatic hernia											1 (1.00)	

**Fig. 4:**
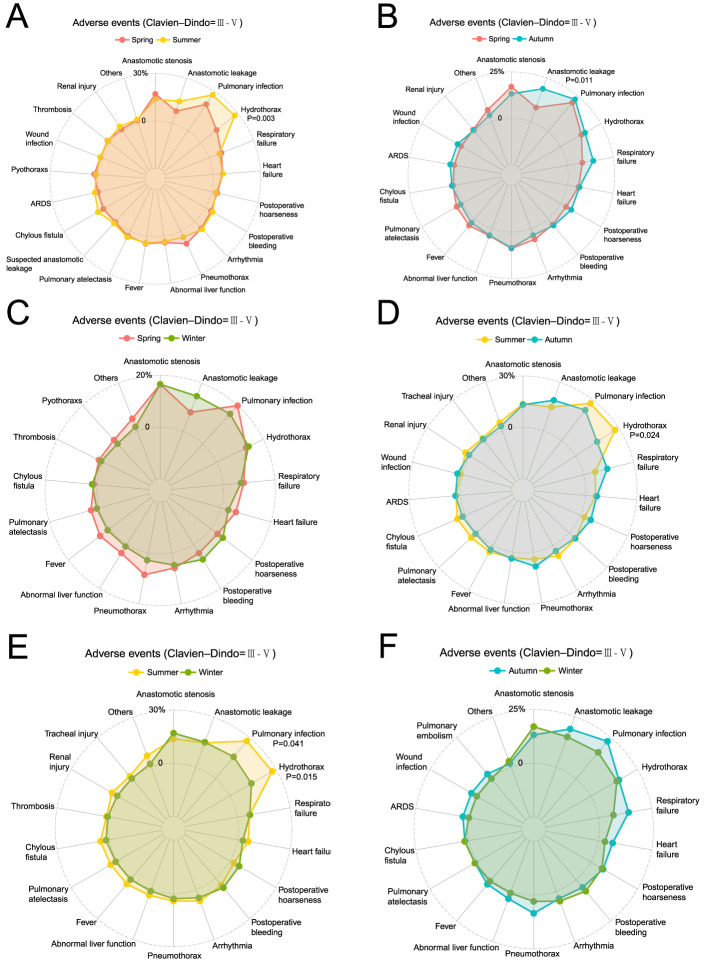
Adverse events of participants with Clavien-Dindo grade III-V. **A** Adverse events of Spring Group and Summer Group before PSM; **B** Adverse events of Spring Group and Autumn Group before PSM; **C** Adverse events of Spring Group and Winner Group before PSM; **D** Adverse events of Summer Group and Autumn Group before PSM; **E** Adverse events of Summer Group and Winner Group before PSM; **F** Adverse events of Autumn Group and Winner Group before PSM

**Fig. 5:**
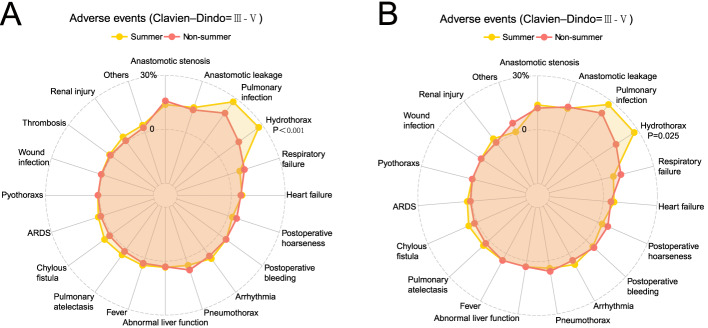
**A** Adverse events of Summer Group and Non-Summer Group before PSM; **B** Adverse events of Summer Group and Non-Summer Group after PSM

Analysis of specific complication types revealed significant seasonal variations. The Summer Group showed a significantly higher incidence of hydrothorax compared to other groups (P < 0.05). The Summer Group also exhibited a higher rate of pulmonary infection than other groups, with the difference being statistically significant when compared to the Winter Group (P = 0.041). The Autumn Group demonstrated a significantly higher incidence of anastomotic leakage compared to the Spring Group (P = 0.011) (Fig. 4). Following PSM, the Summer Group continued to show a significantly higher incidence of hydrothorax compared to the non-Summer groups (P = 0.025) (Table 4 and Fig. 5).

**Table 4:** Adverse events (Clavien–Dindo ≥ III, 2009)

	Before PSM						After PSM					
Adverse events	Summer (n = 116)			Not summer (n = 353)			Summer (n = 116)			Not summer (n = 116)		
	III	IV	V	III	IV	V	III	IV	V	III	IV	V
Anastomotic stenosis	16(13.79%)			55(15.58%)	1(0.28%)		16(13.79%)			13(11.21%)	1(0.86%)	
Anastomotic leakage	11(9.48%)	6(5.17%)		32(9.07%)	14(3.97%)	1(0.28%)	11(9.48%)	6(5.17%)		14(12.07%)	4(3.45%)	
Pulmonary infection	17(14.66%)	15(12.93%)		34(9.63%)	34(9.63%)	2(0.57%)	17(14.66%)	15(12.93%)		15(12.93%)	10(8.62%)	
Hydrothorax	32(27.59%)			49(13.88%)			32(27.59%)			18(15.52%)		
Respiratory failure	1(0.86%)	7(6.03%)		2(0.57%)	30(8.50%)	2(0.57%)	1(0.86%)	7(6.03%)		2(1.72%)	11(9.48%)	
Heart failure	5(4.31%)	2(1.72%)		12(3.40%)	6(1.70%)	1(0.28%)	5(4.31%)	2(1.72%)		3(2.59%)	2(1.72%)	
Postoperative hoarseness	3(2.59%)			15(4.25%)	3(0.85%)		3(2.59%)			6(5.17%)	1(0.86%)	
Postoperative bleeding	4(3.45%)	2(1.72%)		13(3.68%)	5(1.42%)		4(3.45%)	2(1.72%)		4(3.45%)	3(2.59%)	
Arrhythmia	7(6.03%)	1(0.86%)		14(3.97%)	4(1.13%)		7(6.03%)	1(0.86%)		4(3.45%)	1(0.86%)	
Pneumothorax	5(4.31%)			25(7.08%)			5(4.31%)			7(6.03%)		
Abnormal liver function	4(3.45%)			9(2.55%)	2(0.57%)		4(3.45%)			3(2.59%)	1(0.86%)	
Fever	5(4.31%)			11(3.12%)			5(4.31%)			5(4.31%)		
Pulmonary atelectasis	5(4.31%)			7(1.98%)			5(4.31%)			3(2.59%)		
Suspected anastomotic leakage	2(1.72%)			1(0.28%)			2(1.72%)					
Chylous fistula	3(2.59%)	3(2.59%)		4(1.13%)	2(0.57%)		3(2.59%)	3(2.59%)		2(1.72%)		
ARDS		3(2.59%)			4(1.13%)			3(2.59%)			1(0.86%)	
Pyothoraxs		1(0.86%)		2(0.57%)	1(0.28%)			1(0.86%)		1(0.86%)		
Wound infection		1(0.86%)		3(0.85%)	1(0.28%)			1(0.86%)			1(0.86%)	
Pulmonary embolism				1(0.28%)	1(0.28%)	1(0.28%)						
Delirium				1(0.28%)	1(0.28%)					1(0.86%)		
Thrombosis	2(1.72%)			4(1.13%)			2(1.72%)					
Ketosis		1(0.86%)						1(0.86%)				
Renal injury	3(2.59%)	1(0.86%)		2(0.57%)	1(0.28%)		3(2.59%)	1(0.86%)			1(0.86%)	
Tracheal injury		2(1.72%)				1(0.28%)		2(1.72%)				
Cerebral infarction				1(0.28%)								
Gastric perforation					1(0.28%)							
Diaphragmatic hernia					1(0.28%)

### Risk factors

**Fig. 6:**
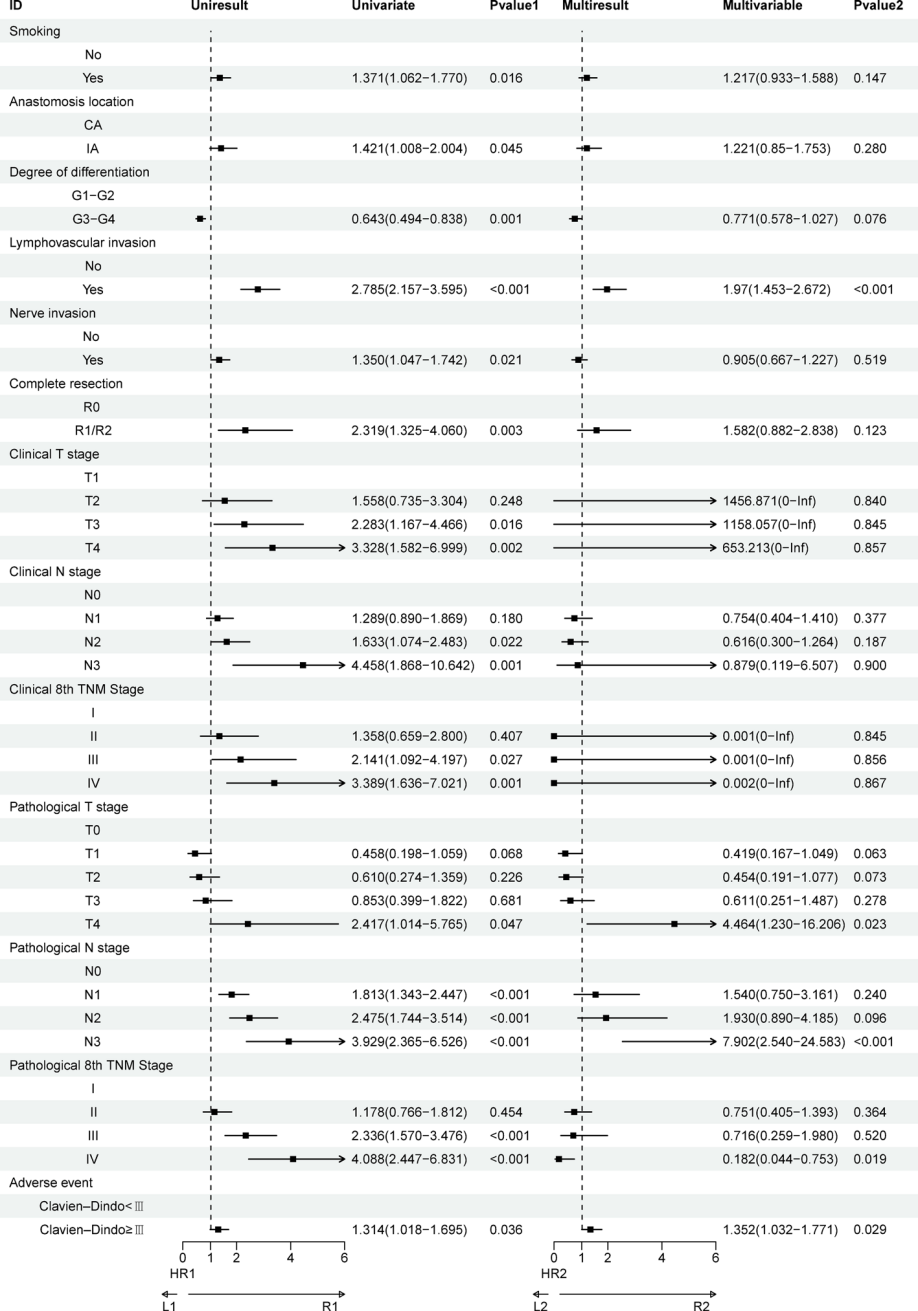
Univariate and multivariate Cox regression analyses regarding factors affecting OS of patients

Univariate analysis revealed that smoking status, anastomosis location (intrathoracic vs. cervical anastomosis), lymphovascular invasion, nerve invasion, R0 resection status, occurrence of adverse events, and tumor stage/grade were all associated with OS. Multivariate Cox proportional hazards regression analysis identified lymphovascular invasion (P < 0.001), pT4 stage (P = 0.023), pN3 stage (P < 0.001), pTNM stage IV (P = 0.023), and the occurrence of severe adverse events (Clavien-Dindo ≥ III) (P = 0.029) as independent prognostic factors for OS (Fig. 6).

**Fig. 7:**
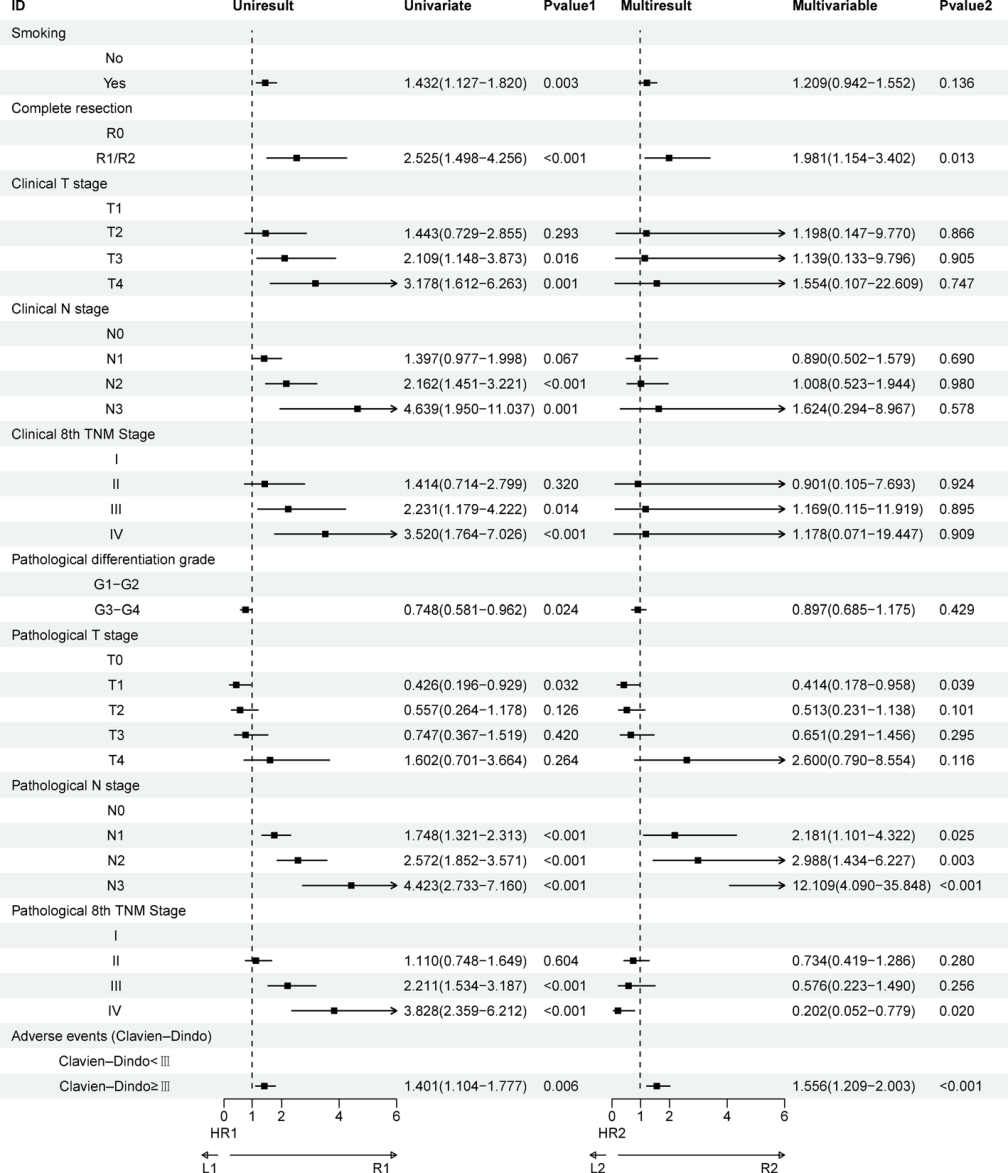
Univariate and multivariate Cox regression analyses regarding factors affecting DFS of patients

Similarly, univariate analysis for DFS showed associations between smoking status, R0 resection status, adverse events, and tumor stage/grade. Multivariate analysis confirmed R0 resection status (P = 0.013), pN3 stage (P = 0.003), pN3 stage (P < 0.001), pTNM stage IV (P = 0.020), and severe adverse events (Clavien-Dindo ≥ III) (P < 0.001) as independent predictors of DFS (Fig. 7).

## Discussion

This retrospective cohort study investigates the impact of seasonal variation on postoperative outcomes in elderly patients with ESCC undergoing esophagectomy. The analysis of short-term outcomes reveals significant seasonal variations in specific postoperative complications. The summer group demonstrates a significantly higher incidence of hydrothorax and pulmonary infection compared to other groups. These findings highlight the potential influence of environmental factors on the risk profile of specific postoperative complications. The increased incidence of hydrothorax and pulmonary infection during the summer may be linked to physiological changes associated with elevated temperatures. These seasonal variations in complication rates, despite not impacting overall survival, emphasize the importance of individualized patient management and the potential need to adjust perioperative strategies according to the time of year. The long-term outcomes suggest that the timing of surgery may not serve as a major independent predictor of long-term survival in this specific patient population. The spring group consistently shows numerically longer median OS, DFS, RMST, and RMDFST compared to other seasons, although these differences do not reach statistical significance.

With the ongoing trend of global aging, the treatment of elderly patients with ESCC has become an increasingly important focus [14]. Compared to younger patients, elderly individuals often exhibit reduced physiological reserves and heightened sensitivity to environmental factors, such as temperature fluctuations and seasonal changes. This study further corroborates this observation, particularly highlighting the increased susceptibility of elderly ESCC patients to postoperative complications during specific seasons, notably the summer. As shown in our findings, patients undergoing surgery in the summer experienced a significantly higher incidence of hydrothorax and pulmonary infection. These complications may be linked to the physiological stress induced by warmer temperatures, which could affect immune function, wound healing, and overall recovery [25]. Despite these seasonal variations in short-term complications, the long-term survival outcomes, including OS and DFS, were not significantly influenced by the timing of surgery. This suggests that, while environmental factors may play a role in postoperative recovery, the season in which surgery is performed is not a major independent predictor of long-term survival in elderly ESCC patients.

While inpatient wards and operating rooms are generally maintained at stable temperature and humidity levels, patients are not continuously confined to these controlled environments throughout their entire perioperative course. In practice, pre- and postoperative imaging studies, endoscopies, and other diagnostic tests often require patients to leave the ward and traverse corridors or radiology suites where air handling may differ, and doors frequently open to the outside. Early ambulation protocols typically include brief walks in outdoor hospital areas or semi-open spaces (e.g., courtyards, balconies), exposing patients to ambient temperature and humidity. Family visits and short excursions to hospital cafeterias or clinics similarly introduce patients to seasonal fluctuations in weather. Following discharge—particularly during the initial weeks—patients’ recovery continues at home, where ambient conditions (temperature fluctuations, humidity, pollen levels, and temperature-related changes in air quality) vary by season. These intermittent exposures to the external environment, combined with patients’ reduced physiological reserves and compromised immune function, could plausibly contribute to the seasonal differences observed in rates of hydrothorax, pulmonary infection, and anastomotic leakage.

Moreover, our analysis indicated that the incidence of anastomotic leakage was significantly higher in autumn compared to spring (P = 0.011). However, no statistically significant differences were observed in the rates of anastomotic leakage between other seasons. Given this lack of consistent differences across seasons, we did not conduct further analyses.

Currently, there was still a lack of individualized treatment approach tailored specifically for elderly ESCC patients. Similar to the general recommendations for ESCC, elderly patients are advised to undergo a multimodal treatment regimen, primarily focusing on surgery, combined with preoperative neoadjuvant therapies to reduce tumor burden and improve resectability. Postoperative adjuvant therapies, such as chemotherapy, radiotherapy, or immunotherapy, are then chosen based on the patient’s physical condition, pathological results, and any complications that arise [5, 6, 34]. However, our study underlines the importance of adopting a more personalized approach for elderly patients.

This study has several limitations that should be taken into account when interpreting the findings. Firstly, the retrospective design inherently introduces potential selection bias, as the study relied on pre-existing data, limiting control over confounding variables despite the use of PSM. Secondly, while seasonal variation was the primary focus, the study did not account for intra-seasonal environmental factors such as air quality, humidity, and temperature fluctuations, which may also influence postoperative outcomes. Finally, the impact of unmeasured factors like preoperative nutritional status, psychological conditions, or specific perioperative care strategies could not be fully addressed. Future multi-center, prospective studies with larger sample sizes are necessary to confirm these findings and explore underlying mechanisms for seasonal influences on esophagectomy outcomes.

## Conclusion

This study found no significant impact of seasonal variations on overall survival and disease-free survival in elderly patients with esophageal squamous cell carcinoma undergoing esophagectomy. While certain complications varied by season, such as increased hydrothorax and pulmonary infections in summer, these did not affect long-term survival outcomes.

## Data Availability

The datasets supporting the results of the present study can be obtained from the corresponding author upon reasonable request.

## Abbreviations

ESCC: Esophageal squamous cell carcinoma
EC: Esophageal cancer
OS: Overall surviva
DFS: Disease-Free Survival
RMST: Restricted mean survival time
RMDFST: Restricted Mean Disease-Free Survival Time
HRs: Hazard ratios
CIs: Confidence intervals
BMI: Body mass index
WHO: World Health Organization
PSM: Performing propensity score matching
KPS: Karnofsky Performance Status
UICC/AJCC: Union for International Cancer Control/American Joint Committee on Cancer
SCCH-ECCM Database: Sichuan Cancer Hospital & Institute Esophageal Cancer Case Management Database
